# Autocatalytic Oxidization of Nanosilver and Its Application to Spectral Analysis

**DOI:** 10.1038/srep03990

**Published:** 2014-02-05

**Authors:** Guiqing Wen, Yanghe Luo, Aihui Liang, Zhiliang Jiang

**Affiliations:** 1 Key Laboratory of Ecology of Rare and Endangered Species and Environmental Protection of Ministry Education, Guangxi Normal University, Guilin 541004, China

## Abstract

The stable yellow nanosilver (AgNP) and blue nanosilver (AgNPB) sols were prepared by the NaBH_4_ procedure. The new nanocatalytic reaction of AgNP-NaCl-H_2_O_2_ was investigated by surface plasmon resonance (SPR) absorption, resonance Rayleigh scattering (RRS), surface-enhanced Raman scattering (SERS) and scanning electron microscope (SEM) techniques. The autocatalytic oxidization of Ag on AgNP surface by H_2_O_2_ was observed firstly and the AgNP/AgCl nanoparticles were characterized. The [Ag^+^] from AgNP is different to the Ag^+^ from AgNO_3_ that adsorb on the AgNP surface. An autocatalytic oxidization mechanism was proposed to explain experimental phenomena. The relationship between the SPR absorption peaks and the RRS peaks of AgNPB was studied, and three characteristic RRS peaks called as out-of-plane quadrupole, out-of-plane dipole and in-plane dipole RRS peaks were observed firstly. Using AgNP as nanoprobe, a simple, sensitive and selective RRS method was developed for assay of H_2_O_2_ in the range of 2.0 × 10^−8^-8.0 × 10^−5^ mol/L.

Nanoparticles have become research hot-spot in subjects such as physics, chemistry and materials due to their unique physical and chemical properties^1^,^2^,^3^,^4^,^5^. Based on the optical properties of nanoparticles, some new types of optical sensors and optoelectronic devices have been developed^6^,^7^,^8^,^9^. And the research of nanocatalysis and its application has become the important research directions in catalytic synthesis and nanoanalysis^10^,^11^,^12^,^13^,^14^,^15^,^16^. Among the nanocatalysis, the reports were mainly about nanogold catalysis and nano titanium dioxide photocatalysis^17^,^18^,^19^. Recently, some new nanocatalytic analytical methods were established^20^,^21^,^22^,^23^,^24^,^25^, based on the catalysis of nanogold on the Cu(II)-glucose, HAuCl_4_-ascrobic, HAuCl_4_-citrate, Te(IV)-Sn(II), phosphomolybdic acid-formic acid and Ag(I)-hydroquinone particle reactions. It is rare report about nanosilver catalytic reaction. In synthesis of triangular nanosilver^26^,^27^, the catalysis of nanosilver has been observed. Nanosilver modified by aptamer exhibited strong catalysis of the Cu_2_O particle reaction of Fehling reagent-glucose, and it has been used to determine trace melamine by RRS technique^28^. Up to date, the autocatalytic oxidization of AgNP and its application in trace H_2_O_2_ analysis have not been reported.

The content of H_2_O_2_ is closely related to the photochemical reactions, oxidation-reduction reactions in natural water bodies. It is one of the important factors to affect the transfer, transformation, and ecological effect of chemical substances in water, and it is also one of the main reasons in the formation of acid rain^29^. In addition, the detection of H_2_O_2_ content is also very important in biochemical reactions, clinical test, food safety, and other fields^20^,^21^,^22^,^23^,^24^,^25^,^26^,^27^,^28^,^29^,^30^,^31^,^32^. At present, the spectral methods for H_2_O_2_ content are mainly including chemiluminescence, fluorescence, spectrophotometry, etc^33^,^34^,^35^. Among them, spectrophotometry is commonly used for its simple operation and low-cost. Recently, stabile metal nanoparticles, especially nanogold and nanosilver, are interesting to analyst. The spectral probe, based on nanogold color and RRS, have been used for determination of tumor markers, Hg^2+^, H_2_O_2_, HCl and so on^36^,^37^,^38^,^39^,^40^,^41^. Compare to nanogold, AgNP has the advantage of low cost, and its aggregates do not interfere to the absorbance measurement since its absorption is very weak. In addition, stabile AgNP can be prepared by modern synthesis procedure and it has become novel spectral probe due to its strong SPR absorption, strong RRS and SERS effects of its aggregates^42^. It has been utilized to colorimetric chiral recognition of enantiomers, detection of DNA and melamine etc^28^,^43^,^44^,^45^. However, there is no report about the research and application of AgNP-NaCl-H_2_O_2_ autonanocatalytic system yet. In this paper, the reaction mechanism of AgNP-NaCl-H_2_O_2_ was studied by SPR, RRS^46^,^47^,^48^, SERS and SEM techniques. Thus, two simple, rapid and sensitive spectral methods have been developed to determine H_2_O_2_.

## Results

### Scanning electron microscope

**Figure 1:**
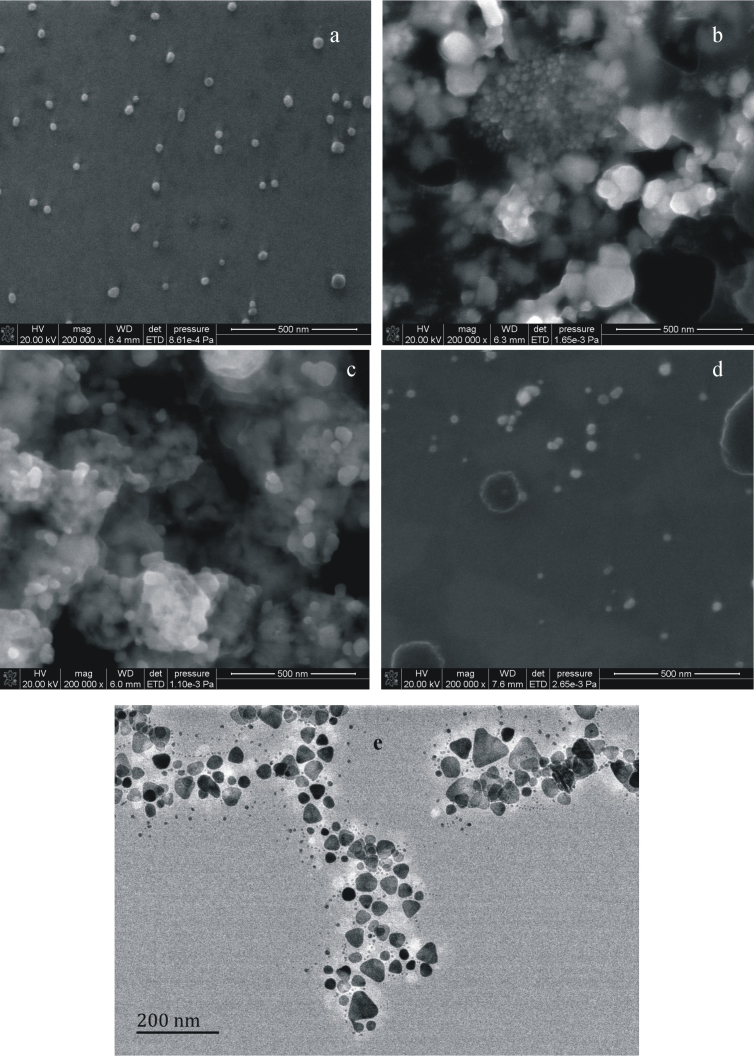
SEM (a–d) and TEM (e) of AgNP. (a): 9.25 × 10^−5^ mol/L AgNP; (b):9.25 × 10^−5^ mol/L AgNP-2.0 × 10^−3^ mol/L NaCl-20 × 10^−5^ mol/L H_2_O_2_; (c): 9.25 × 10^−5^ mol/L AgNP-2.0 × 10^−3^ mol/L H_2_SO_4_-2.0 × 10^−3^ mol/L NaCl-3.75 × 10^−5^ mol/L FeSO_4_-1.5 × 10^−5^ mol/L H_2_O_2_; (d): 5.0 × 10^−5^ mol/L AgNPB; (e): 5.0 × 10^−5^ mol/L AgNPB.

**Figure 2:**
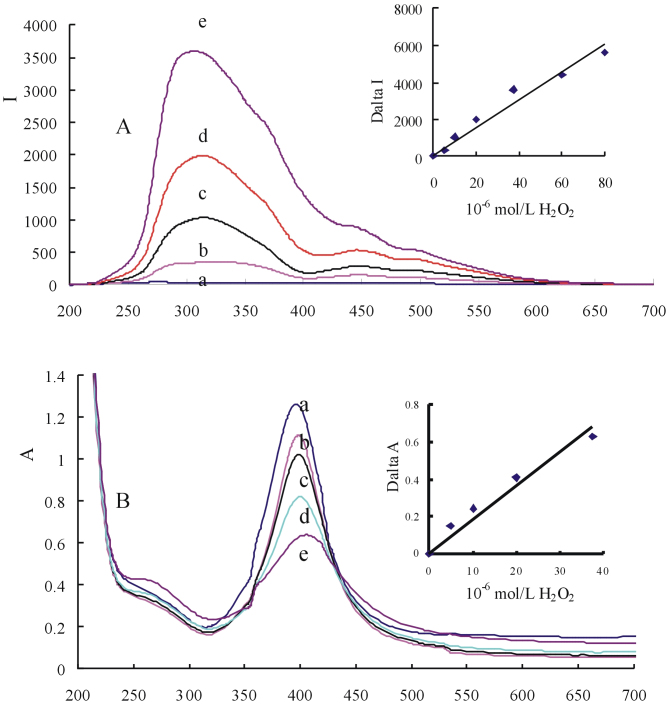
RRS (A) and SPR (B) spectra of AgNP-NaCl-H_2_O_2_ system. (a): 9.25 × 10^−5^ mol/L AgNP −2.0 × 10^−3^ mol/L NaCl; (b):a-5.0 × 10^−6^ mol/L H_2_O_2_; (c): a-1.0 × 10^−5^ mol/L H_2_O_2_; (d): a-2.0 × 10^−5^ mol/L H_2_O_2_; (e): a-5.0 × 10^−5^ mol/L H_2_O_2_.

Stabile AgNP sol was prepared by NaBH_4_ reduction of AgNO_3_ in the presence of citrate. The SEM shows that they are spherical, with average size of 10 ± 2 nm (Fig. 1a). In AgNP-NaCl-H_2_O_2_ system, there is an autocatalytic oxidation reaction on the surface of AgNP to generate large Ag_n_/AgCl particles with an average size of 60 ± 15 nm (Fig. 1b). In AgNP-H_2_SO_4_-NaCl-FeSO_4_-H_2_O_2_ system, on one hand the autocatalytic oxidation reaction of AgNP generate Ag^+^ on the surface, on the other hand surface atoms of AgNP also can generate Ag^+^ by the Fenton oxidation reaction, so the large Ag_n_/AgCl particles with an average size of 75 ± 16 nm was formed (Fig. 1c). SEM of AgNPB system shows that they are nearly spherical, with particle size between 6–100 nm and an average size of 40 ± 8 nm (Fig. 1d). The shape of AgNPB can not be observed satisfactorily by SEM, and the TEM of AgNPB system was done. Figure 2e indicated that there triangle nanosilver particles in the system, with the side length between 30–90 nm and an average side length of 45 ± 10 nm, in addition to the nearly spherical particles.

### RRS spectra

In 2.0 × 10^−3^ mol/L NaCl medium, AgNPs are stabile and its RRS signal is very weak (Fig. 2A). With addition of H_2_O_2_, AgNP catalyze H_2_O_2_ to produce HO**·** and oxidize Ag atoms on the surface of AgNP to produce [Ag^+^]. The [Ag^+^] combined with Cl^−^ to form [AgCl] with strong hydrophobicity and then lead to form larger AgNP/AgCl aggregates that obviously enhanced the RRS intensity at 330 nm, 460 nm and 500 nm. The most sensitive RRS peak at 330 nm was selected to use in this paper. In existence of Fe(II) and H_2_SO_4_, the system had four RRS peaks at 290 nm, 360 nm, 455 nm and 500 nm (Fig. 1S). Fe(II) can hydrolyze and cause weak aggregation of AgNPs, and the blank increased. When adding H_2_O_2_, the Fenton reaction (FeSO_4_-H_2_O_2_) also produced HO**·** that oxidize AgNP to form [Ag^+^] and AgNP/[AgCl] particles. With H_2_O_2_ concentration increase its peak enhanced linearly due to more particles forming.

**Figure 3:**
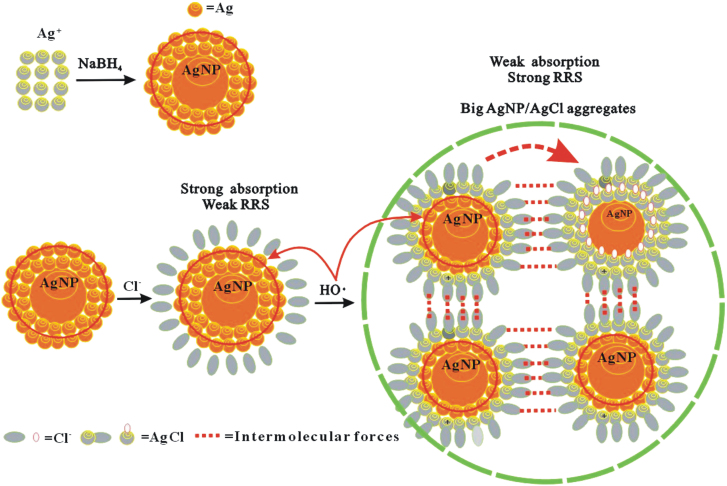
Principle of autocatalytic oxidization of AgNP to detect H_2_O_2_.

**Figure 4:**
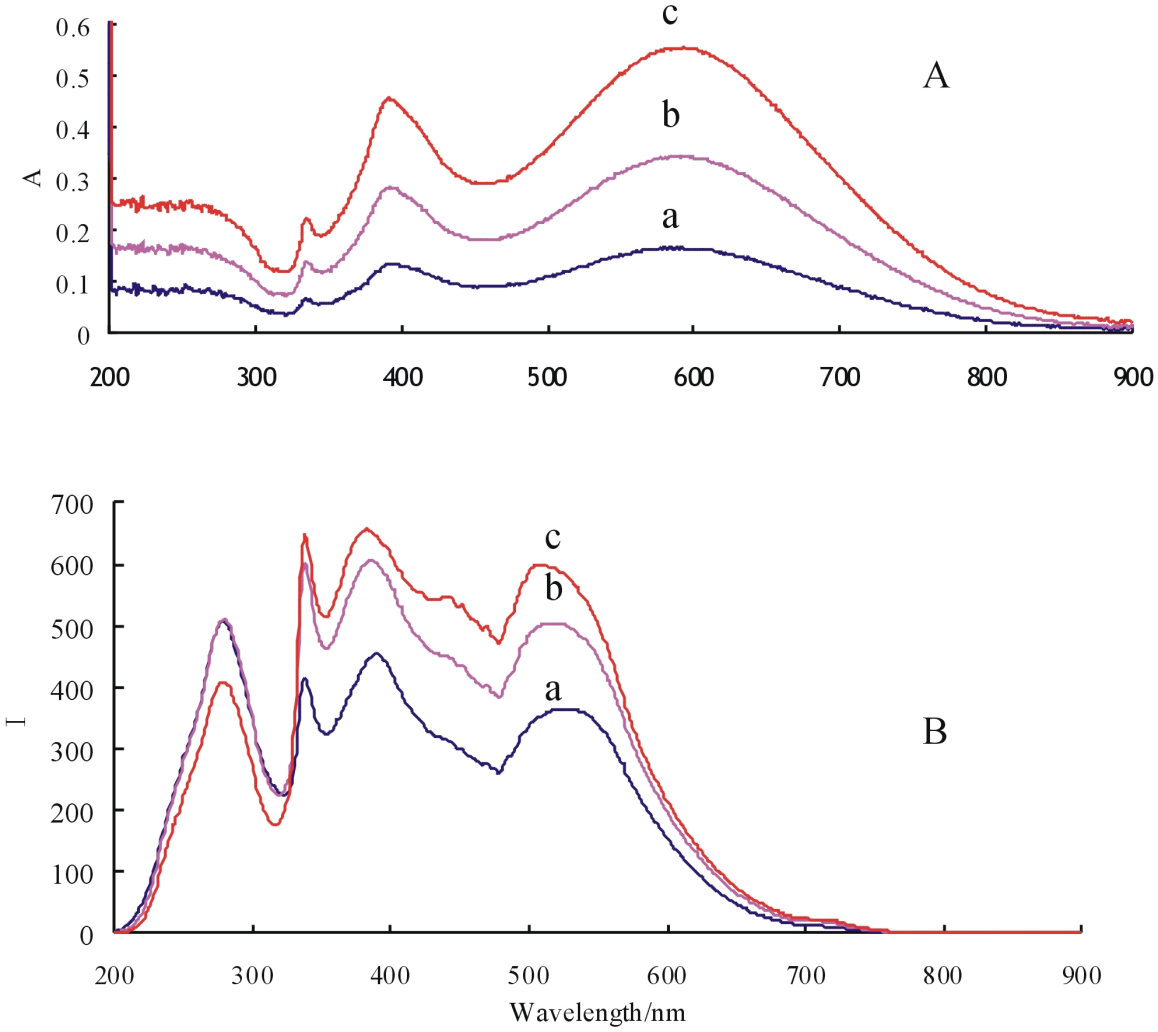
SPR spectra (A) and RRS spectra (B) of AgNPB. (a): 2.5 × 10-5 mol/L AgNPB; (b): 5.0 × 10-5 mol/L AgNPB; (c): 7.5 × 10-5 mol/L AgNPB.

With addition of different concentration of AgNO_3_ to the system of 2.0 × 10^−3^ mol/L NaCl-0.035% sodium citrate, AgCl particles were generated and exhibited strong scattering signal at 335 nm (Fig. 2S). The increased intensity Δ*I_335 nm_* was linear to AgNO_3_ concentration in the range of 12.5–100 × 10^−6^ mol/L with the regression equation of Δ*I_335 nm_* = 53.3c_Ag+_ - 93. For the system of 2.0 × 10^−3^ mol/L NaCl-0.035% sodium citrate-2.0 × 10^−3^ mol/L H_2_SO_4_-3.75 × 10^−5^ mol/L FeSO_4_-AgNO_3_, AgCl particles exhibited strong scattering signal at 335 nm (Fig. 3S). The increased intensity Δ*I_335 nm_* was linear to AgNO_3_ concentration in the range of 12.5-100 × 10^−6^ mol/L with the regression equation of Δ*I_335 nm_* = 51.0c_Ag+_ + 116. This suggests that RRS signal's enhancement of AgNP-NaCl-H_2_O_2_ system is the result of the formation of AgCl particles. When adding different concentration of Ag^+^ to the AgNP-NaCl-sodium citrate system, the RRS spectrum (Fig. 4S) is different with that of AgNP-NaCl-H_2_O_2_ and the former is weaker. It also suggests that [Ag^+^] which produced by AgNP surface oxidation is different with that adsorption on the surface of the AgNP by adding AgNO_3_. Compare to RRS spectra of NaCl-sodium citrate-AgNO_3_ system, the RRS intensity of AgNP-NaCl-sodium citrate system is greatly reduced and has a valley at 395 nm, as the result of the strongest absorption of AgNPs at 395 nm.

### SPR absorption spectra

Mie theory^49^ pointed out that, spherical nanoparticles have only one SPR absorption peak. Spherical AgNP with diameter of 20–30 nm has the strongest SPR peak near 400 nm^50^, which is out-of-plane dipole SPR absorption peak^51^. In the systems of NaCl and NaCl-H_2_SO_4_-FeSO_4_, both have an AgNP SPR absorption peak at 395 nm (Fig. 2B, Fig. 5S). The absorbance at 395 nm of the two systems decreased linearly with the H_2_O_2_ concentration increased and can be chosen to determine H_2_O_2_. The AgNO_3_-NaCl and AgNO_3_-NaCl-H_2_SO_4_-FeSO_4_ systems were examined by spectrophotometry. With addition of different AgNO_3_ concentration to the two systems of 2.0 × 10^−3^ mol/L NaCl-0.035% sodium citrate and 2.0 × 10^−3^ mol/L NaCl-0.035% sodium citrate −2.0 × 10^−3^ mol/L H_2_SO_4_ −3.75 × 10^−5^ mol/L FeSO_4_, the produced AgCl particles exhibited weak SPR peak at 285 nm (Fig. 6S,7S). The absorbance increased slowly with the AgNO_3_ concentration increased in the range of 12.5-100 × 10^−6^ mol/L because AgCl particles have weak absorption. In the medium of 5.0 × 10^−4^ mol/L NaCl, AgNPB has two SPR absorption peaks at 330 nm and 530 nm (Fig. 8S). The absorbance at 530 nm decreased linearly with the H_2_O_2_ concentration increased in the range of 2–8 × 10^−5^ mol/L H_2_O_2_ that can be used to determine H_2_O_2_.

### SERS spectra of AgNP-NaCl-H_2_O_2_ system

SERS technology is very sensitive for the detection of nano-aggregate, and it is very important to choice a suitable molecular probe. Reportedly cationic dye rhodamine 6G was used as a sensitive SERS probe^52^, but it can interact with AgNP to form aggregate and cannot be used in the analysis of AgNP-NaCl system. Victoria blue B (VBB), used as a SERS probe, had very weak Raman signals in the two systems of 9.25 × 10^−5^ mol/L AgNP and 9.25 × 10^−5^ mol/L AgNP-2.0 × 10^−3^ mol/L NaCl. With addition of H_2_O_2_, SERS signals enhanced due to the formation of AgCl and Ag/AgCl aggregate and the system exhibited Raman peaks at 224 cm^−1^, 307 cm^−1^, 351 cm^−1^, 564 cm^−1^, 608 cm^−1^, 772 cm^−1^, 1127 cm^−1^, 1179 cm^−1^, 1309 cm^−1^, 1359 cm^−1^, 1508 cm^−1^, 1571 cm^−1^, 1647 cm^−1^ (Fig. 9S). This demonstrated that there are AgNP/AgCl aggregates in the system.

## Discussion

### Mechanism of autocatalytic oxidization of AgNP

Stabile AgNP in size of 10 nm was prepared conveniently by NaBH_4_ reduction of Ag^+^. When NaCl was added, Cl^−^ can be adsorbed on the surface of AgNP, and the signals of SPR absorption and RRS are still very weak that indicated no aggregation in the system. After adding H_2_O_2_, small AgNP can catalyze H_2_O_2_ to produce free radicals HO**·** with strong oxidation ability, which can oxidize Ag atoms on the surface of AgNP to produce [Ag^+^] that is different with the Ag^+^ from AgNO_3_. The [Ag^+^] combined with Cl^−^ to form [AgCl] molecules which had strong hydrophobic property and then lead to form large AgNP/AgCl aggregates that enhanced the scattering signal. Small size HO**·** can penetrate the gap of [AgCl] molecules to further oxidize Ag atoms on inner layer of AgNP (Fig. 3), and made AgNP become smaller and its SPR absorption weaker. When H_2_O_2_ increased, the RRS intensity increased linearly due to more big AgNP/AgCl aggregates forming, and the SPR absorption decreased linearly due to much less small nanosilver forming. Thus, two new SPR absorption and RRS methods were established to determine H_2_O_2_.

According to the generation mechanism of HO**·** and the autocatalytic oxidation mechanism of AgNP^53^,^54^ (AgNP = Ag_n_ = Ag_m + 2k_), the main reactions of AgNP-NaCl-H_2_O_2_ system are as follows,
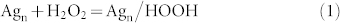

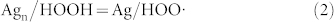

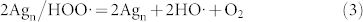

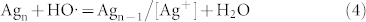

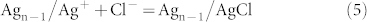
In the presence of NaCl, the reducing ability of Ag was enhanced and made the reaction of H_2_O_2_ oxidize AgNP to form Ag_m-2k_/[AgCl]_2k_ complex particles become easily, as the result of the formation of AgCl particle with low solubility. The total reaction is as follow,
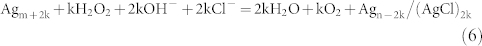


### Relationship between the SPR absorption peaks and the RRS peaks of AgNPB

AgNPBs exhibited special optical property that have one sharp out-of-plane quadrupole SPR absorption peak at 330 nm (Fig. 4A), one out-of-plane dipole peak at 390 nm, and one broad in-plane dipole peak at 580 nm^54^,^55^. The absorbance of the three peaks was linearly increased with AgNPB concentration increased. The study of RRS spectra of nanoparticles in liquid phase shown that, their RRS peaks are closely related with the emission intensity of light source and the SPR absorption peaks^56^,^57^. The light source of model F-7000 Hitachi fluorescence spectrometer has the strongest emission wavelength at 280 nm that cause a scattering peak at 280 nm, and the emission intensity weaken as the increase of the wavelength. AgNPB has a sharp scattering peak at 330 nm which is corresponding to the out-of-plane quadrupole SPR absorption peak (Fig. 4B) that was called as out-of-plane quadrupole RRS peak. AgNPB has a strong RRS peak at 390 nm that was ascribed to out-of-plane dipole SPR absorption, was called as out-of-plane dipole RRS peak. Besides, the in-plane dipole peak at 530 nm is violet-shift 50 nm compare to its SPR absorption peak because the light source has strong emission at 530 nm. Though the RRS intensity of the three peaks increased with AgNPB concentration increased, they had no linear relationship since the sols exist in multiple scattering.

### Analytical features

Under the selected conditions (Fig. 10S–19S), the RRS intensity at 330 nm (*I*) of different H_2_O_2_ concentration was recorded, and the working curve between Δ*I* and H_2_O_2_ concentration was drawn. The linear range of AgNP-NaCl system was 2.0 × 10^−8^-8.0 × 10^−5^ mol/L, with a regression equation of Δ*I* = 71.4c-1.6, a correlation coefficient of 0.9852, and a detection limit of 8 × 10^−9^ mol/L. A 5.0 × 10^−7^ mol/L, 5.0 × 10^−6^ mol/L and 20 × 10^−6^ mol/L H_2_O_2_ were determined five times, and the related standard deviations (RSD) were 4.1%, 3.7% and 3.8% respectively, this showed that the RRS method has good accuracy. The linear range of AgNP-NaCl-H_2_SO_4_-FeSO_4_ system was 1.0 × 10^−7^-2.5 × 10^−5^ mol/L, with a detection limit of 2 × 10^−8^ mol/L. In the RRS analytical system, the AgNP-NaCl is most sensitive, simple, stabile and low blank (Table 1S), and it was chosen to detect H_2_O_2_ concentration. The SPR methods of the two systems also can be used to determine H_2_O_2_ with low-cost, though they were not as sensitive as RRS methods. According to the procedure, a standard solution containing 20 × 10^−6^ mol/L H_2_O_2_ and various coexistent compounds were examined, with a relative error of less than ± 10%. A 100 times of ClO_4_^−^ and SO_4_^2−^, 70 times of Ca(II) and Mg(II), 50 times of Cu^2+^, Mo^6+^, I^−^, triethanolamine, Co^2+^, NO_2_^−^, 10 times of Mn^2+^, Br^−^, citric acid, and 2.5 mg/L HSA did not interfere with the determination. This indicated that the method has good selectivity.

## Methods

### Apparatus and reagents

A Model F-7000 fluorescence spectrophotometer (Hitachi Company, Japan) was used to record the RRS spectra by means of synchronous scanning excitation wavelength λ_ex_ and emission wavelength λ_em_ (λ_ex_-λ_em_ = Δλ = 0) and the RRS intensity. A Model TU-1901 double beams spectrophotometer (Puxi Tongyong Apparatus Limited Company, Beijing) was used to record the SPR spectra and the SPR intensity. A model JSM-6380LV scanning electron microscope (Electronic Stock Limited Company, Japan), a model of JEM-2100F field emission transmission electron microscope (Electronic Stock Limited Company, Japan), a model DXR smart Raman spectrometer (Thermo Fisher Scientific Co., Ltd., USA), a moldel SK8200LH ultrasonic reactor (Kedao Company, Shanghai, China), and a model magnetic stirrer (Zhongda Instrumental Plant, Jiangsu, China) were used.

A 1.0 × 10^−3^ mol/L AgNO_3_ solution, 1% (W/V) sodium citrate solution, 0.05 mol/L NaCl solution, and 1.0 × 10^−3^ mol/L FeSO_4_ solution were used. A 0.05% (W/V) NaBH_4_ was prepared freshly. A 0.100 mol/L H_2_O_2_ standard solution was prepared as follows: 1.02 mL H_2_O_2_ (30%) was diluted to 100 mL with water, it was standardized by KMnO4 procedure, and was diluted to 5.00 × 10^−4^ mol/L before use.

A 1.85 × 10^−4^ mol/L AgNP was prepared as follows: 9.25 mL 1.0 × 10^−3^ mol/L AgNO_3_ and 3.5 mL 1% trisodium citrate were added into a conical flask with stirring and diluted to 40 mL with water, then 4 mL 0.05% NaBH_4_ was added slowly with about 15 min, the mixture was diluted to 50 mL, and it can be used after 24 h to make the NaBH_4_ decomposing completely. The preparation of AgNP sols was repeated five times, the SPR peak is at 395 nm with an average absorption value of 2.50 ± 0.10, and the sols were stabile within 20 days (Fig.20S). A 1.0 × 10^−4^ mol/L AgNPB was prepared as follows: 40 mL of water, 500 μL 1.0 × 10^−2^ mol/L AgNO_3_, 1.5 mL 6.0 × 10^−2^ mol/L sodium citrate, 120 μL 30% H_2_O_2_, and 200 μL 0.1 mol/L NaBH_4_ were added into a triangle flask in turn with constantly stirring for 15 min. Then heat to boil for 5 min to get rid of the excess H_2_O_2_ and the solution was diluted to 50 mL. All reagents were of analytical grade and the water was highly pure sub-boiling water.

### Procedure

A 1.0 mL 1.85 × 10^−4^ mol/L AgNP solution, 80 μL 0.05 mol/L NaCl (or adding 80 μL 5.0 × 10^−2^ mol/L H_2_SO_4_, 75 μL 1.0 × 10^−3^ mol/L FeSO_4_), and a certain amount of H_2_O_2_ solution were added into a 5 mL calibrated tube in turn, then diluted to 2 mL and mixed well. The RRS intensity at 330 nm (*I*) was recorded by a fluorescence spectrophotometer with synchronous scanning (*λ*_ex_-*λ*_em_ = Δ*λ* = 0). A blank (*I*_0_) without H_2_O_2_ was recorded and the value of Δ*I = I–I*_0_ was calculated.

## Author Contributions

G.Q.W. and Y.H.L. performed the experiment and measurement analysis. G.Q.W. and Z.L.J. prepared Fig.1–4. A.H.L. and Z.L.J. contributed to the discussion and measurement analysis. All authors contributed to the preparation of the manuscript and reviewed the manuscript.

## Supplementary Material

Supplementary Information SUPPLEMENTARY INFO
