# Supplementary material for: Autocatalytic Oxidization of Nanosilver and Its Application to Spectral Analysis
Source: Sci Rep. 2014 Feb 5;4:3990. doi: 10.1038/srep03990 (PMC3913915; doi:10.1038/srep03990)
Supplement: Supplementary Information — SUPPLEMENTARY INFO [file srep03990-s1.doc]

**Autocatalytic Oxidization of Nanosilver and Its Application to Spectral Analysis**

Guiqing Wen, Yanghe Luo, Aihui Liang^*^, Zhiliang Jiang^*^

(Key Laboratory of Ecology of Rare and Endangered Species and Environmental Protection of Ministry Education, Guangxi Normal University, Guilin 541004, China)

I

f

e

d

c

b

a

Wavelength/nm

Fig. 1S RRS spectra of AgNP-H_2_SO_4_-NaCl-FeSO_4_-H_2_O_2_ system

a: 9.25×10^-5^ mol/L AgNP-2.0×10^-3^ mol/L H_2_SO_4_-2.0×10^-3^ mol/L NaCl-3.75×10^-5^ mol/L FeSO_4_; b:a-2.5×10^-6^ mol/L H_2_O_2_; c: a-5.0×10^-6^ mol/L H_2_O_2_; d: a-1.0×10^-5^ mol/L H_2_O_2_; e: a-1.5×10^-5^ mol/L H_2_O_2_; f: a-2.0×10^-5^ mol/L H_2_O_2_.

g

f

e

d

c

b

a

I

Wavelength/nm

Fig. 2S RRS spectra of NaCl-sodium citrate-AgNO_3_ system

a: 2.0×10^-3^ mol/L NaCl-0.035% sodium citrate; b:a-1.25×10^-5^ mol/L AgNO_3_; c: a-2.5×10^-5^ mol/L AgNO_3_; d: a-3.75×10^-5^ mol/L AgNO_3_; e: a-5.0×10^-5^ mol/L AgNO_3_; f: a-7.5×10^-5^ mol/L AgNO_3_; g: a-1.0×10^-4^ mol/L AgNO_3_.

g

f

e

d

c

b

a

I

Wavelength/nm

Fig. 3S RRS spectra of NaCl-sodium citrate-H_2_SO_4_-FeSO_4_-AgNO_3_ system

a: 2.0×10^-3^ mol/L NaCl-0.035% sodium citrate -2.0×10^-3^ mol/L H_2_ SO_4_ -3.75×10^-5^ mol/L FeSO_4_ ; b:a-1.25×10^-5^ mol/L AgNO_3_; c: a-2.5×10^-5^ mol/L AgNO_3_; d: a-3.75×10^-5^ mol/L AgNO_3_; e: a-5.0×10^-5^ mol/L AgNO_3_; f: a-7.5×10^-5^ mol/L AgNO_3_; g: a-1.0×10^-4^ mol/L AgNO_3_.

g

f

e

d

c

b

a

Wavelength/nm

Fig. 4S RRS spectra of AgNP-NaCl- sodium citrate -AgNO_3_ system

a: 9.25×10^-5^ mol/L AgNP-2.0×10^-3^ mol/L NaCl-0.035% sodium citrate; b:a-1.25×10^-5^ mol/L AgNO_3_; c: a-2.5×10^-5^ mol/L AgNO_3_; d: a-3.75×10^-5^ mol/L AgNO_3_; e: a-5.0×10^-5^ mol/L AgNO_3_; f: a-7.5×10^-5^ mol/L AgNO_3_; g: a-1.0×10^-4^ mol/L AgNO_3_.

a

b

c

d

e

f

g

A

Wavelength/nm

Fig. 5S SPR spectra of AgNP-H_2_SO_4_-NaCl-FeSO_4_-H_2_O_2_ system

a: 9.25×10^-5^ mol/L AgNP -2.0×10^-3^ mol/L H_2_SO_4_-2.0×10^-3^ mol/L NaCl -3.75×10^-5^ mol/L FeSO_4_; b:a-2.5×10^-6^ mol/L H_2_O_2_; c: a-5.0×10^-6^ mol/L H_2_O_2_; d: a-1.0×10^-5^ mol/L H_2_O_2_; e: a-1.5×10^-5^ mol/L H_2_O_2_; f: a-2.0×10^-5^ mol/L H_2_O_2_; g: a-2.5×10^-5^ mol/L H_2_O_2_.

A

g

a

Wavelength/nm

Fig. 6S SPR spectra of NaCl-sodium citrate-AgNO_3_ system

a: 2.0×10^-3^ mol/L NaCl-0.035% sodium citrate; b:a-1.25×10^-5^ mol/L AgNO_3_; c: a-2.5×10^-5^ mol/L AgNO_3_; d: a-3.75×10^-5^ mol/L AgNO_3_; e: a-5.0×10^-5^ mol/L AgNO_3_; f: a-7.5×10^-5^ mol/L AgNO_3_; g: a-1.0×10^-4^ mol/L AgNO_3_.

A

g

a

Wavelength/nm

Fig. 7S SPR spectra of NaCl- sodium citrate -H_2_SO_4_-FeSO_4_-AgNO_3_ system

a: 2.0×10^-3^ mol/L NaCl-0.035% sodium citrate -2.0×10^-3^ mol/L H_2_ SO_4_ -3.75×10^-5^ mol/L FeSO_4_ ; b:a-1.25×10^-5^ mol/L AgNO_3_; c: a-2.5×10^-5^ mol/L AgNO_3_; d: a-3.75×10^-5^ mol/L AgNO_3_; e: a-5.0×10^-5^ mol/L AgNO_3_; f: a-7.5×10^-5^ mol/L AgNO_3_; g: a-1.0×10^-4^ mol/L AgNO_3_.

A

a

b

c

d

e

Wavelength/nm

Fig. 8S SPR spectra of AgNPB-NaCl-H_2_O_2_ system

a: 5.0×10^-5^ mol/L AgNPB-5.0×10^-4^ mol/L NaCl; b: a-2.0×10^-5^ mol/L H_2_O_2_; c: a-4.0×10^-5^ mol/L H_2_O_2_; d: a-6×10^-5^ mol/L H_2_O_2_; e: a-8×10^-5^ mol/L H_2_O_2_.

Raman shift/cm^-1^

I

c

b

a

Fig. 9S SERS spectra of AgNP-NaCl-H_2_O_2_-VBB system

a: 9.25×10^-5^ mol/L AgNP-2.0×10^-3^ mol/L NaCl-1.0×10^-5^ mol/L VBB; b:a-5.0×10^-6^ mol/L H_2_O_2_; c: a-1.0×10^-5^ mol/L H_2_O_2_.

**Optimization of conditions**

The effect of AgNP concentration on Δ*I* was studied (Fig. 10S). The Δ*I* value increased linearly with the AgNP concentration increasing in the range of 0-5.0×10^-5^ mol/L due to more AgNP/AgCl aggregates forming. When the concentration of AgNP is 9.25×10^-5^ mol/L, the system has the maximum of Δ*I*. So 9.25×10^-5^ mol/L AgNP was chosen.

ΔI

*µ*M AgNP

Fig. 10S Effect of AgNP concentration on Δ*I*

2.0×10^-3^ mol/L NaCl-2.5×10^-5^ mol/L H_2_O_2_.

The effect of NaCl concentration on Δ*I* was studied (Fig. 11S). The Δ*I* value increased with the NaCl concentration increasing in the range of 0-1.0×10^-3^ mol/L due to the formation of more [AgCl] and AgNP/AgCl particles. The system has the maximum of Δ*I* when the concentration of NaCl is 2.0×10^-3^ mol/L. More than that, the Δ*I* decreased since AgNP aggregated or precipitated. So 2.0×10^-3^ mol/L NaCl was chosen. The effect of NaF, NaBr and NaI on Δ*I* was studied too, and the result shown that the three kinds of halogen ions had no enhancement effect like Cl^-^.

ΔI

mM NaCl

Fig. 11S Effect of NaCl concentration on Δ*I*

9.25×10^-5^ mol/L AgNP-2.5×10^-5^ mol/L H_2_O_2_.

Fenton reagent (Fe^2+^/H_2_O_2_) is a kind of easy-access hydroxyl radical antioxidants. In this paper, acid-FeSO_4_ was introduced into the system to improve the sensitivity. The effect of H_2_SO_4_ concentration on Δ*I* was studied in the presence of FeSO_4_ (Fig. 12S). The system has the maximum of Δ*I* when the concentration of H_2_SO_4_ is 2.0×10^-3^ mol/L. So 2.0×10^-3^ mol/L H_2_SO_4_ was chosen. In addition, the effect of HNO_3_, HClO_4_, and H_3_BO_3_ was examined and the result shown that their sensitization effects were close to that of H_2_SO_4_. The effect of FeSO_4_ concentration on Δ*I* was studied (Fig. 13S). The system has the maximum of Δ*I* when the concentration of FeSO_4_ is 5.0×10^-5^ mol/L. So 5.0×10^-5^ mol/L FeSO_4_ was chosen.

The effect of reaction time on the absorbance and Δ*I* was studied respectively. The absorbance and Δ*I* of AgNP-NaCl-H_2_O_2_ system reached the maximum and remained stable when the reaction time is more than 15 min (Fig. 14, 15S). The absorbance and Δ*I* of AgNP-NaCl-H_2_SO_4_- FeSO_4_-H_2_O_2_ system reached the maximum and remained stable when the reaction time is more than 40 and 15 min, respectively (Fig. 16S, 17S). This suggests that the three methods of SPR, RRS of AgNP-NaCl-H_2_O_2_ system and RRS of AgNP-NaCl-H_2_SO_4_-FeSO_4_-H_2_O_2_ system to detect H_2_O_2_ is rapid and do not need consider the effect of reaction time.

A

a

**b**

t/min

Fig. 12S Effect of reaction time on Absorbance of AgNP-NaCl-H_2_O_2_ system

a:9.25×10^-5^ mol/L AgNP-2.0×10^-3^ mol/L NaCl;b: a-2.5×10^-5^ mol/L H_2_O_2_

b

a

ΔI

t/min

Fig. 13S Effect of reaction time on Δ*I* of AgNP-NaCl-H_2_O_2_ system

a:9.25×10^-5^ mol/L AgNP-2.0×10^-3^ mol/L NaCl;b: a-2.0×10^-5^ mol/L H_2_O_2_

The effect of reaction temperature on the absorbance and Δ*I* was studied. The ΔA of AgNP-NaCl-H_2_O_2_ and AgNP-NaCl-H_2_SO_4_-FeSO_4_-H_2_O_2_ systems were maximum and remained stable when the temperature was in the range of 20-40℃ (Fig. 18Sc, 18Sf). The ΔA decreased rapidly when the reaction temperature exceeded 40℃. The A value of AgNP-NaCl-H_2_SO_4_-FeSO_4_-H_2_O_2_ system increased when the reaction temperature exceeded 40℃ (Fig. 18Sd). Possibly because the hydrolysis of Fe^2+^ led to AgNPs coagulation, and the phenomenon agreed with that of the RRS signal decreased with the temperature raise. By the effect experiments of reaction temperature on RRS intensity (Fig. 19S), AgNP-NaCl system for determination of H_2_O_2_ wasn’t affected by temperature almostly.

ΔI

mM H_2_SO_4_
Fig. 14S Effect of H_2_SO_4_ concentration on Δ*I*

9.25×10^-5^ mol/L AgNP**-**2.0×10^-3^ mol/L NaCl **-**5.0×10^-5^ mol/L FeSO_4_ -2.5×10^-5^ mol/L H_2_O_2_.

µM

ΔI

Fig. 15S Effect of FeSO_4_ concentration on Δ*I*

9.25×10^-5^ mol/L AgNP-2.5×10^-3^ mol/L H_2_SO_4_**-**2.0×10^-3^ mol/L NaCl **-**5.0×10^-5^ mol/L FeSO_4_ -2.5×10^-5^ mol/L H_2_O_2_.

A

a

b

t/min

Fig. 16S Effect of reaction time on Absorbance of AgNP-NaCl-H_2_SO_4_- FeSO_4_-H_2_O_2_ system

a:9.25×10^-5^ mol/L AgNP-2.5×10^-3^ mol/L H_2_SO_4_-2.0×10^-3^ mol/L NaCl-5.0×10^-5^ mol/L FeSO_4_ ;b: a-1.0×10^-5^ mol/L H_2_O_2_

ΔI

b

a

t/min

Fig. 17S Effect of reaction time on Δ*I* of AgNP-NaCl-H_2_SO_4_- FeSO_4_-H_2_O_2_ system

a:9.25×10^-5^ mol/L AgNP-2.5×10^-3^ mol/L H_2_SO_4_-2.0×10^-3^ mol/L NaCl-5.0×10^-5^ mol/L FeSO_4_ ;b: a-1.0×10^-5^ mol/L H_2_O_2_

**a**

**d**

**b**

**f**

**e**

**c**

**A**

T/℃

Fig. 18S Effect of reaction temperature on the absorption value

a:9.25×10^-5^ mol/L AgNP-2.0×10^-3^ mol/L NaCl; b:9.25×10^-5^ mol/L AgNP-2.5×10^-3^ mol/L NaCl-1.5×10^-5^ mol/L H_2_O_2_;c: a-b; d:9.25×10^-5^ mol/L AgNP-2.5×10^-3^ mol/L H_2_SO_4_-2.0×10^-3^ mol/L NaCl-5.0×10^-5^ mol/L FeSO_4_;e:9.25×10^-5^ mol/L AgNP-2.5×10^-3^ mol/L H_2_SO_4_-2.0×10^-3^ mol/L NaCl-5.0×10^-5^ mol/L FeSO_4_ -1.5×10^-5^ mol/L H_2_O_2_;f:d-e.

**e**

**f**

**b**

**c**

**d**

**a**

I

T/℃

Fig. 19S Effect of reaction temperature on I

a:9.25×10^-5^ mol/L AgNP-2.0×10^-3^ mol/L NaCl; b: a+1.5×10^-5^ mol/L H_2_O_2_;c: b - a; d:9.25×10^-5^ mol/L AgNP-2.5×10^-3^ mol/L H_2_SO_4_-2.0×10^-3^ mol/L NaCl-5.0×10^-5^ mol/L FeSO_4_; e: d+1.5×10^-5^ mol/L H_2_O_2_; f: e-d.

Days

A_395 nm_

Wavelength/nm

Fig.20S UV-Vis spectrum of the 1.85×10^-4^ mol/L AgNP sol

Table 1S Analysis features of AgNP systems for the determination of H_2_O_2_

| System | Regression equation | LR(µmol/L) | Coefficient | DL(µmol/L) |
| --- | --- | --- | --- | --- |
| AgNP-NaCl | Δ*I* =71.4c +1.6 | 0.02-80 | 0.9852 | 0.008 |
|  | ΔA =0.0155c+0.0527 | 2.5-37.5 | 0.9834 | 1.0 |
| AgNP-NaCl-H_2_SO_4_-FeSO_4_ | Δ*I* =133.2c-62.5 | 0.1-25 | 0.9934 | 0.02 |
|  | ΔA =0.0394c+0.004 | 1.0-20 | 0.9734 | 0.5 |
| AgNP-NaCl-FeSO_4_ | Δ*I* =37.7c-26.6 | 0.5-100 | 0.9967 | 0.05 |
| AgNP-NaCl-HNO_3_ | Δ*I* =35.2c-43.8 | 1.6-70 | 0.9962 | 0.8 |
| AgNP-NaCl-HClO_4_ | Δ*I* =35.9c-32.6 | 0.6-60 | 0.9988 | 0.2 |
| AgNP-NaCl-HBO_3_ | Δ*I* =31.6c-1.7 | 0.4-65 | 0.9978 | 0.2 |

**Analysis of samples**

Waste water was taken and filtrated, then, H_2_O_2_ content in 100μL water sample was detected according to the procedure. The known quantity H_2_O_2_ was added and the total H_2_O_2_ content was determined (Table 2S). The recovery was in the range of 99 -102%.

**Table 2**S Analytical results of water samples (n=3)

| Sample | H_2_O_2_ content  /(μmol.L^-1^) | Added  /(μmol.L^-1^) | Total found  /(μmol.L^-1^) | Recovery /% |
| --- | --- | --- | --- | --- |
| 1 | 15.3 | 5.0 | 20.4 | 102 |
| 2 | 9.5 | 10.0 | 19.6 | 101 |
| 3 | 2.2 | 15.0 | 17.0 | 99 |
